# Long-Term Outcomes, Moderators, and Predictors in Online Mindfulness–Based Cognitive Therapy for People With Cancer: Secondary Analysis of a Randomized Controlled Trial

**DOI:** 10.2196/79928

**Published:** 2026-04-17

**Authors:** Nasim Badaghi, Jelle Lubbers, Judith Prins, Saskia Kelders, Anne Speckens, Linda Kwakkenbos

**Affiliations:** 1 Department of Psychiatry, Radboud University Medical Center, Geert Grooteplein Zuid 10, Nijmegen, 6525 GA, The Netherlands, 024 361 1111; 2 Department of Psychology, Health and Technology, University of Twente, Twente, The Netherlands; 3 Optentia Research Focus Area, North-West University, Vanderbijlpark, South Africa; 4 Department of Clinical Psychology, Behavioural Science Institute, Radboud University, Nijmegen, The Netherlands

**Keywords:** telehealth, cancer, clinical trials, long-term effect, mindfulness, oncology

## Abstract

**Background:**

A 3-armed randomized controlled trial (RCT) demonstrated that 2 formats of online mindfulness–based cognitive therapy (eMBCT)—group-blended and individual-unguided—effectively reduced psychological distress and improved positive health outcomes in people with cancer and survivors, when compared to care as usual, up to 3 months posttreatment. However, the long-term effectiveness and possible predictors and moderators of treatment outcomes remain unknown.

**Objective:**

This study examined the long-term effects (up to 9 months posttreatment) of group-blended and individual-unguided eMBCTs on psychological distress and other mental health outcomes in people with cancer. Additionally, it explored possible predictors and moderators of treatment effects across the 2 delivery formats.

**Methods:**

The study population consisted of people with cancer who were initially randomized to group-blended or individual-unguided eMBCT within a 3-arm RCT, augmented by those who completed the waitlist condition and were subsequently randomly allocated to one of the eMBCT formats. Both groups were assessed over a 9-month follow-up period. Outcomes completed at baseline, posttreatment, and 3-, 6-, and 9-month follow-up assessments included psychological distress (primary), fear of cancer recurrence, rumination, fatigue, mindfulness skills, decentering, self-compassion, and well-being. Linear mixed-effects models examined changes over time, while linear mixed-effects models and binary logistic regression analyzed potential predictors and moderators of psychological distress and dropout.

**Results:**

Of the 186 participants enrolled in the 3-arm RCT, 161 participants were randomly assigned to either group-blended or individual-unguided eMBCTs after adding those initially assigned to the waiting condition (group-blended: n=71; individual-unguided: n=90). The majority of participants were female (n=129, 80%), diagnosed with breast cancer (n=78, 48%), and were undergoing or had completed treatment with curative intent (n=124, 77%). The mean age was 52.8 (SD 11.4) years. Both eMBCT formats resulted in significant reductions in psychological distress, fear of cancer recurrence, rumination, and fatigue, alongside improvements in mindfulness skills, decentering, and self-compassion, up to 9 months posttreatment. Higher baseline rumination, as well as lower mindfulness skills and self-compassion at baseline, predicted larger reductions in psychological distress from baseline to the 9-month follow-up period. Additionally, highly distressed participants in the group-blended eMBCT arm were more prone to dropout than those with lower distress scores, whereas psychological distress was not associated with dropout in the individual-unguided format. No other significant moderators were identified.

**Conclusions:**

Group-blended and individual-unguided eMBCTs effectively reduced psychological distress and improved well-being among people with cancer and survivors, with greater benefits for those with fewer psychological resources. However, individuals experiencing higher levels of distress were more likely to discontinue group-blended eMBCT. These findings highlight the importance of considering individual preferences and pragmatic factors in treatment decisions. Larger, fully powered RCTs are needed to confirm these results and provide more definitive guidance on treatment format selection.

## Introduction

### Background

Cancer can strongly affect a person’s life, beginning at the moment of diagnosis and possibly lasting a lifetime. Although the growing number of cancer survivors reflects improvements in medical progress [1], it also presents a challenge: more people must live with the long-term consequences of the disease. Research indicates that people with cancer can experience detrimental psychological effects that may persist after completing cancer treatment, significantly impacting the quality of life and mental health of cancer survivors for years [2]. In addition, it has been shown that long-term cancer survivors (≥5 y after diagnosis) experience reduced quality of life, fatigue, and neuropsychological issues, such as memory and attention deficits [3].

Mindfulness-based interventions (MBIs) have proven to be effective in alleviating the psychological challenges encountered by people with cancer [4,5]. There is robust evidence showing that, in oncological settings, MBIs are not only effective in symptom reduction [4,5] but also in fostering positive health outcomes such as mindfulness skills, self-compassion, and personal growth [6]. At the core of MBIs lies mindfulness, which is defined as the awareness that emerges from paying attention to the present moment [7]. MBIs help people recognize their emotions, thoughts, bodily sensations, and automatic reactions with nonjudgmental awareness and kindness, allowing them to respond in a more adaptive and beneficial way. MBIs are traditionally delivered through in-person group sessions, typically lasting around 2.5 hours, and led by a trained mindfulness teacher.

While MBIs have been shown to be effective for people with cancer, attending face-to-face group sessions can be challenging due to travel demands and limited scheduling flexibility [8]. To address these barriers, online MBIs (eMBIs) have been developed, showing strong evidence for reducing distress, depressive symptoms, stress, and sleep problems, while improving quality of life [9-11]. However, little is known about their long-term impact, or possible predictors and moderators of treatment outcomes in different eMBI formats.

### Long-Term Effects

According to a recent meta-analysis by Chayadi et al [12], MBIs can significantly reduce symptoms of depression (Hedges *g*=0.43), anxiety (Hedges *g*=0.55), and cancer-related fatigue (Hedges *g*=0.43) in oncology settings up to at least 3 months post-MBI. In addition, at longer follow-ups (6 months on average), MBIs also demonstrate statistically significant effects on psychological distress, anxiety, depression, and fatigue, with effect sizes ranging from Hedges *g*=0.19 to 0.36 [4]. Research on the long-term effects of eMBIs, however, remains relatively scarce. Current evidence does not yet clarify whether the benefits of eMBIs are sustained over extended periods [11]. Given the increasing prevalence of cancer survivors and the growing demand for more flexible and accessible interventions, establishing the long-term effectiveness of eMBIs is highly relevant.

### Predictors and Moderators

Although MBIs or eMBIs have been shown to be beneficial, not everyone may benefit in the same way. Understanding these differences is essential for tailoring interventions to meet personal needs and enhance their effectiveness. Research on predictors and moderators can provide valuable insights into determining what works best for whom. A predictor is a variable that forecasts an outcome regardless of intervention condition, while a moderator influences the strength or direction of the relationship between a predictor and an outcome. Prior research found that lower baseline levels of psychological distress, rumination, and neuroticism, as well as higher levels of extraversion and agreeableness, predicted lower levels of distress at a 9-month follow-up after face-to-face or online mindfulness–based cognitive therapy (eMBCT), a specific form of eMBI [13]. Additionally, individuals who were less mindful and conscientious benefited more from therapist-assisted eMBCT than face-to-face MBCT [13]. However, the results were derived from 1 study only. Predictors and moderators of more scalable eMBIs such as individual-unguided formats remain unidentified. Recent studies highlight the need to explore these variables to enhance the effectiveness and accessibility of eMBIs for people with cancer [10,14].

### Current Research

We recently completed a 3-arm randomized controlled trial (RCT) that evaluated the effects of 2 formats of eMBCT (ie, group-blended and individual-unguided) on psychological distress in people with cancer [15]. At posttreatment, only the group-blended eMBCT significantly reduced psychological distress. However, at the 3-month follow-up, both intervention formats appeared to be effective in comparison to care as usual (CAU). Additionally, both formats led to reductions in rumination and improvements in mindfulness skills and decentering, whereas only the group-blended eMBCT also increased self-compassion at the 3-month follow-up.

This study aimed to examine the long-term effects of group-blended and individual-unguided eMBCTs on individuals with cancer over the course of the 9-month follow-up. Specifically, we aimed to explore the effects of both formats on psychological distress, fear of cancer recurrence, fatigue, rumination, mindfulness skills, decentering, self-compassion, and well-being.

Additionally, we investigated potential predictors and moderators that may influence treatment effects. Among the predictors and moderators examined, this study focused on rumination, mindfulness, decentering, and self-compassion. A recent scoping review of mediators of MBIs identified mindfulness skills, decentering, and mindfulness-related attitudes (such as self-compassion) as the most consistent mechanisms linking MBIs to improvements in mental health and well-being across adult clinical and nonclinical populations [16]. These mechanisms primarily explained reductions in symptoms, such as stress, anxiety, and depression, and to a lesser extent improvements in well-being [16]. In people with cancer, rumination has also been shown to mediate MBI effects, including in the trial underlying the follow-up study [17,18], and different components of mindfulness skills have been identified as mediators in this population [18]. Additionally, individuals with lower baseline mindfulness skills appear to benefit more from therapist-assisted eMBCT than from traditional MBCT in the long term [13]. Decentering has likewise been proposed as a mediator of mindfulness in people with cancer [19] and may help counteract rumination by offering a protective mechanism. Together, these factors are important for understanding how individual characteristics interact with different intervention formats.

## Methods

Follow-up data from a 3-arm, parallel RCT were used [15]. Initially, participants were randomized to group-blended eMBCT, individual-unguided eMBCT, or CAU. After completing their 3-month CAU follow-up period, participants in the CAU condition were randomized to either group-blended or individual-unguided eMBCT.

### Ethical Considerations

This study was approved by the ethical review board of Radboud University Medical Center (Central Committee on Research Involving Human Subjects Arnhem-Nijmegen; NL73117.091.20), registered in the Dutch Registry CCMO (NL73117.091.20) and on ClinicalTrials.gov (NCT05336916). We published a protocol in advance of trial completion [15] and conducted an uncontrolled feasibility study before running the full RCT [20]. Eligible participants were sent comprehensive written information about the study and an informed consent form via email and regular mail. Once the signed consent form was returned, participants were enrolled in the trial and received a link via email to complete baseline assessments. All participants provided informed consent prior to participation. Their privacy and confidentiality were fully protected, and no compensation was provided for their participation.

### Participants

Because the methods used in studies based on our RCT data are similar, we have reused some methodological descriptions from previous publications [15,20]. In doing so, we followed the guidelines provided by the Text Recycling Research Project [21]. Participants were recruited between January 2021 and September 2023 via posters in health care settings, flyers handed out by health care professionals, posts in regional newspapers, online posts placed on websites of cancer-related organizations, and social media platforms. Eligible participants were adults who had been diagnosed with cancer (irrespective of type or stage of cancer and time since diagnosis), had internet access, the ability to use a computer, and a good command of the Dutch language. Participants were excluded from the study if they had previously attended more than 4 sessions of any MBI, had a severe psychiatric comorbidity, drug or alcohol dependence, significant cognitive impairment, or were unwilling to be randomized to 1 of the 3 study conditions. Participants could continue any ongoing medical, psychological, or paramedical care during the study, except for participation in other MBIs outside the study context.

### Procedure

The study was conducted at the Radboudumc Expertise Center for Mindfulness in Nijmegen, The Netherlands. Interested participants could reach out via email, phone, or the contact form on the trial website. Eligible participants were sent comprehensive written information about the study and an informed consent form via email and regular mail. Once the signed consent form was returned, participants were enrolled in the trial and received a link via email to complete baseline assessments. Baseline assessments consisted of online self-reported questionnaires, including demographic characteristics and various patient-reported outcome measures. After participants completed the questionnaires, they were randomized into one of three conditions (group-blended, individual-unguided, or CAU) by an independent research assistant using the secure data management platform Castor EDC. Randomization was stratified based on 2 variables: cancer type (breast cancer vs other types) and treatment intent (curative vs palliative).

For participants in the CAU group who were randomized a second time, the same procedure as the first randomization in the RCT was followed. After completing the CAU period, a research assistant contacted them by email or phone to ask about their willingness to participate in eMBCT. If they agreed, they completed the T2 outcome assessments (new baseline) and were then randomized to either group-blended or individual-unguided eMBCT. While this rerandomization allowed for comparisons between the 2 eMBCT interventions, it is important to note that there is no CAU control group after the 3-month follow-up period.

### Intervention

#### Overview

The group-blended and individual-unguided eMBCT programs were adaptations of the MBCT protocol developed by Segal et al [22]. The intervention formats followed the standard 8-week MBCT structure, with weekly 2.5-hour sessions incorporating formal and informal mindfulness practices, reflective exercises, daily home practice (30 min to 1 h), and a silent day. The program content was adapted to include psychoeducation on mindfulness for cancer and grief, and the movement exercises were modified to accommodate people with cancer. Detailed descriptions of the intervention content, development process, and conditions have been published elsewhere [15].

#### Intervention Delivery

Participants could access the eMBCT program in a private section of the Minddistrict platform (Minddistrict Development BV), which provided all necessary program information and materials. Participants created accounts and accessed the program through an internet browser on a website or by downloading the application onto their devices. While the session content was consistent across both conditions, the delivery methods differed as follows: (1) group-blended eMBCT, where sessions 1, 3, 5, and 8 were conducted as live group sessions via Zoom (Zoom Communications, Inc), led by a mindfulness teacher; and (2) individual-unguided eMBCT, where all sessions were fully self-guided through the Minddistrict platform, where participants engaged with an avatar rather than a physical teacher.

#### Mindfulness Teachers

The mindfulness teachers in the group-blended condition were 2 health care professionals (a psychologist and a social worker) with expertise in psycho-oncology and several years of experience teaching mindfulness- and compassion-based courses. They met the qualification standards set by the Association of Mindfulness Teachers in the Netherlands and Flanders, which are aligned with the UK Network for Mindfulness-Based Teachers’ criteria (2015) [23]. To safeguard consistency, adherence to the protocol, and high-quality program delivery, the teachers attended regular peer supervision sessions with a senior mindfulness teacher (AS). Additionally, they participated in weekly trial research meetings to stay aligned with the study’s objectives and address any challenges encountered during intervention delivery.

### Outcome Measures

Participants completed online questionnaires at 6 time points: baseline (T0); midtreatment; posttreatment (T1); and 3 (T2), 6 (T3), and 9 (T4) months after treatment [15]. Participants in the CAU condition were randomized into either the group-blended or individual-unguided eMBCT after the 3-month follow-up period (T2). For this study, T2 completed by CAU participants was used as baseline, as it better reflected their present state at the start of the intervention. Therefore, participants in the CAU group completed all the assessments mentioned above, as well as 2 additional ones (T5 and T6), which corresponded to the follow-up period for their intervention. The data analyses include results from all 5 time points—T0-T4 for the intervention group and T2-T6 for the participants in the CAU group, who were rerandomized into either the group-blended or individual-unguided eMBCT after 6 months (T2). For the follow-up analyses, T2 became their new T0 measurement.

The primary outcome measure was psychological distress, assessed using the Hospital Anxiety and Depression Scale [24]. Secondary outcomes included the Fear of Cancer Recurrence Inventory [25], the Rumination subscale of the Rumination and Reflection Questionnaire (RRQ) [26], the Fatigue Severity subscale of the Checklist Individual Strength [27], the Five Facet Mindfulness Questionnaire–Short Form (FFMQ-SF) [28], the Decentering subscale of the Experiences Questionnaire (EQ) [29], the Self-Compassion Scale–Short Form (SCS-SF) [30], and the Mental Health Continuum–Short Form [31]. Detailed information about each questionnaire has been published previously [15].

Several constructs (rumination, mindfulness skills, decentering, and self-compassion) were examined in different analytic roles in this study. When analyzed as secondary outcomes, their repeated measurements across all assessment time points were used to evaluate changes over time following the intervention. In contrast, in the prediction and moderation analyses, only baseline values of these constructs were included as predictors or moderators of treatment dropout and psychological distress trajectories. Time-varying values of these variables were not used as predictors in any model.

### Predictors and Moderators

The selection of predictors and moderators was informed by prior empirical studies [4,13]. We examined predictors and moderators of psychological distress (measured by the Hospital Anxiety and Depression Scale) over a 9-month follow-up period, as well as dropout at posttreatment (defined as completing fewer than 4 sessions). It is important to note that dropout was treated as a dependent variable because the study aimed to explore whether baseline characteristics could predict dropout both generally and in relation to the 2 intervention conditions. The variables tested included sex, age, cancer diagnosis (breast cancer vs other types), treatment intent (palliative vs curative), time since diagnosis, and baseline levels of rumination (RRQ), mindfulness skills (FFMQ-SF), decentering (EQ), and self-compassion (SCS-SF).

### Statistical Analyses

#### Overview

All analyses were conducted in SPSS version 29 (IBM Corp). For the CAU group, baseline scores on the primary and secondary outcomes were substituted with post-CAU scores (corresponding to T2 in the initial 3-armed RCT), as these scores were temporally aligned with the start of the intervention. For all analyses, the data from participants who followed group-blended or individual-unguided eMBCT after CAU were combined with the data from participants who were initially randomized to these 2 conditions. After analyzing baseline differences in participant characteristics and outcome measures across the full sample, and comparing baseline scores between initially randomized participants and those who were rerandomized, the only variable that significantly differed between individuals who completed the primary outcome measure at posttreatment and those who did not was fatigue. Noncompleters reported significantly higher fatigue levels, as shown by elevated Fatigue Severity subscale of the Checklist Individual Strength scores (*P*=.02). This indicates that missingness was related to an observed participant characteristic. Assuming missing at random, linear mixed-effects models with (restricted) maximum likelihood estimation were used, which can accommodate incomplete data when missingness depends on observed variables.

#### Long-Term Effects

We built separate linear mixed-effects models for each intervention condition and dependent variable to explore the long-term effects on our primary and secondary outcomes. These linear mixed-effects models used the scores of the primary (psychological distress) or secondary (fear of cancer recurrence, rumination, fatigue, mindfulness skills, decentering, self-compassion, or well-being) outcomes as the dependent variable. Independent variables included time (baseline, posttreatment, 3-month follow-up, 6-month follow-up, and 9-month follow-up), intervention condition (group-blended or individual-unguided eMBCT), and the time×intervention condition interaction. Time was treated as a continuous variable. Random intercepts and random slopes for participants were added. Furthermore, a heterogeneous first-order autoregressive (ARH[1]) covariance structure was applied to model stronger correlations between measurements taken closer together in time compared to those further apart. Restricted maximum likelihood was used to calculate the estimates [32]. In addition, effect sizes over the 9-month follow-up were calculated using Cohen *d* [33], and according to the conventions suggested by Cohen [33], effect sizes of 0.2, 0.5, and 0.8 were considered small, medium, and large, respectively.

#### Prediction and Moderation

Prediction and moderation analyses of psychological distress were conducted using linear mixed models [34]. The dependent variable was psychological distress over the 9-month follow-up period, measured at 5 time points: baseline, posttreatment, and at 3-, 6-, and 9-month follow-ups. Independent variables included time and intervention condition (group-blended eMBCT vs individual-unguided eMBCT). Baseline characteristics used as predictors and moderators were sex, age, cancer diagnosis, cancer treatment intent, time since diagnosis, psychological distress, rumination (RRQ), mindfulness skills (FFMQ-SF), decentering (EQ), and self-compassion (SCS-SF). To assess moderation, interaction terms between intervention condition, moderator variables, and time were included. Random effects were specified for participants to account for within-subject correlations over time [35]. As the analyses were exploratory, original *P* values uncorrected for multiple comparisons were reported.

We defined intervention dropout as attending fewer than 4 of the 8 sessions, which is consistent with other MBI trials [36-38]. An adequate dose of MBCT is typically considered to be participation in at least 4 of the 8 sessions, which captures those participants who have meaningfully engaged with the program [39]. Typical MBCT attendance rates show that over 85% of participants attend all or nearly all of the sessions, with dropouts generally occurring between the orientation and first group session or within the initial few sessions [39]. This attendance distribution justifies the use of the 4-session threshold, aligning with established practices in the field and maintaining consistency with the main RCT of these data. Prediction and moderation analyses of dropout were conducted using binary logistic regression [40]. The dependent variable was dropout status, and the independent variable was the intervention condition. The same baseline characteristics were used as predictors and moderators. Moderation was assessed by including interaction terms between the intervention condition and each moderator.

For a detailed overview of the analyses conducted, please refer to the statistical analysis plan provided in Multimedia Appendix 1.

It is important to note that no power calculation was conducted for the long-term effects or moderation analyses, and the study’s sample size (N=161), along with substantial dropout, may have limited the statistical power to detect small or moderate effects, particularly for 3-way interactions (time× condition × moderator). Therefore, these analyses should be considered exploratory.

## Results

### Overview

A total of 186 participants were originally randomized to group-blended, individual-unguided, and CAU. Of the 54 participants randomized to CAU, 29 were willing to be rerandomized and were assigned to group-blended (n=14) and individual-unguided (n=15) eMBCTs (Table 1; Multimedia Appendix 2). There were no differences in baseline characteristics between those originally randomized to the 2 formats and those who first completed CAU. Of the 161 participants randomized to group-blended or individual-unguided eMBCT, 48 (30%) dropped out after completing fewer than 4 sessions. Dropout rates were significantly higher in the individual-unguided group (n=37, 41%) compared to the group-blended group (n=11, 15%; *P<.001*).

**Table 1.** Baseline sociodemographic and clinical characteristics (N=161).

Characteristic	All (N=161)	Group-blended (n=71)	Individual-unguided (n=90)
Sex, n (%)			
Female	129 (80.1)	55 (77.5)	74 (82.2)
Male	32 (19.9)	16 (22.5)	16 (17.8)
Age (y), mean (SD)	52.8 (11.4)	54.6 (10.9)	51.4 (11.6)
Diagnosis, n (%)			
Breast cancer	78 (48.4)	33 (46.5)	45 (50)
Blood cancer	14 (8.7)	7 (9.9)	7 (7.8)
Prostate cancer	8 (5.0)	4 (5.6)	4 (4.4)
Intestine cancer	7 (4.3)	3 (4.2)	4 (4.4)
Ovarian cancer	7 (4.3)	3 (4.2)	4 (4.4)
Skin cancer	7 (4.3)	4 (5.6)	3 (3.3)
Colon cancer	4 (2.5)	2 (2.8)	2 (2.2)
Other	36 (22.5)	15 (21.2)	21 (23.3)
Treatment intent, n (%)			
Curative	124 (77.0)	55 (77.5)	69 (76.7)
Palliative	37 (23.0)	16 (22.5)	21 (23.3)
Occupation, n (%)			
Employed	99 (61.6)	42 (59.2)	57 (63.3)
Retired	26 (16.1)	12 (16.9)	14 (15.6)
On disability	16 (9.9)	6 (8.5)	10 (11.1)
Houseman or housewife	10 (6.2)	6 (8.5)	4 (4.4)
Unemployed	5 (3.1)	2 (2.8)	3 (3.3)
Other (not specified)	5 (3.1)	3 (4.2)	2 (2.2)
Level of education, n (%)			
High	97 (60.2)	43 (60.6)	54 (60)
Middle	53 (32.9)	22 (31)	31 (34.5)
Low	7 (4.3)	4 (5.6)	3 (3.3)
Other (not specified)	4 (2.5)	2 (2.8)	2 (2.2)
Time since diagnosis (y), n (%)			
0‐1	3 (1.9)	2 (2.8)	1 (1.1)
3	38 (23.6)	16 (22.5)	22 (24.4)
5	62 (38.5)	26 (36.6)	36 (40)
>5	58 (36)	27 (38)	31 (34.4)
Married or in a relationship, n (%)			
Yes	108 (67.1)	46 (64.8)	62 (68.9)
No	53 (32.9)	25 (35.2)	28 (31.1)

### Long-Term Effects

Results from the separate linear mixed-effects models examining the within-group long-term effects of group-blended and individual-unguided eMBCTs showed that both formats led to significant improvements in all primary and secondary psychological outcomes from baseline to the 9-month follow-up. For every outcome and in both intervention conditions, *P* values were below .05. Table 2 shows the descriptive statistics per outcome and intervention condition across time.

**Table 2.** Descriptive statistics and effects of the interaction between time and intervention condition on primary and secondary outcomes in a linear mixed-effects model.

Outcome measure and time		Group-blended		Individual-unguided		Time × intervention condition (*P* value)
		Score, mean (SD)	Participants, n	Score, mean (SD)	Participants, n	
Psychological distress						.76
T0		15.50 (6.32)	69	15.05 (6.94)	90	
T1		10.94 (5.72)	51	11.52 (7.30)	65	
T2		10.58 (5.97)	51	10.94 (5.52)	66	
T3		10.12 (9.50)	50	10.30 (5.85)	53	
T4		10.88 (6.99)	43	9.72 (4.53)	53	
Fear of cancer recurrence						.19
T0		77.84 (22.82)	69	77.06 (24.04)	90	
T1		69.31 (21.16)	49	70.56 (23.53)	63	
T2		65.98 (19.83)	51	70.17 (23.32)	65	
T3		64.40 (16.92)	50	66.87 (23.48)	52	
T4		64.76 (19.79)	42	67.00 (20.79)	51	
Fatigue severity						.19
T0		37.12 (11.16)	69	35.51 (12.01)	90	
T1		32.37 (11.59)	51	32.35 (12.47)	63	
T2		31.49 (12.74)	51	31.86 (11.03)	65	
T3		29.78 (12.19)	50	30.61 (13.79)	52	
T4		29.19 (11.37)	42	29.94 (12.16)	52	
Rumination						.14
T0		3.42 (0.66)	69	3.27 (0.83)	89	
T1		3.06 (0.72)	49	2.98 (0.77)	62	
T2		3.08 (0.78)	51	2.95 (0.78)	64	
T3		3.01 (0.77)	48	2.87 (0.74)	51	
T4		3.05 (0.81)	41	2.91 (0.77)	49	
Mindfulness skills						.11
T0		78.91 (11.21)	69	78.60 (11.26)	90	
T1		84.79 (10.89)	49	84.86 (12.52)	63	
T2		87.37 (10.75)	51	85.26 (11.50)	65	
T3		87.12 (10.26)	50	86.29 (10.13)	51	
T4		87.64 (10.14)	42	86.26 (10.85)	50	
Decentering						.18
T0		33.12 (6.60)	69	33.45 (6.93)	89	
T1		38.86 (5.76)	49	38.55 (6.34)	62	
T2		38.59 (5.93)	51	38.62 (6.68)	65	
T3		39.16 (5.29)	50	38.14 (6.22)	51	
T4		39.07 (6.41)	41	38.54 (6.28)	50	
Self-compassion						.09
T0		48.49 (11.83)	69	50.61 (15.00)	89	
T1		57.39 (11.46)	49	57.81 (13.12)	62	
T2		56.75 (13.60)	51	57.37 (11.73)	64	
T3		56.77 (11.31)	48	58.00 (11.63)	51	
T4		57.49 (13.42)	41	57.08 (12.47)	49	
Well-being						.65
T0		2.89 (0.86)	69	2.87 (0.92)	90	
T1		3.39 (0.74)	49	3.10 (0.93)	63	
T2		3.25 (0.94)	51	3.12 (0.94)	65	
T3		3.26 (0.91)	50	3.09 (0.89)	52	
T4		3.25 (1.01)	42	3.04 (0.94)	51	

Group-blended eMBCT resulted in significant reductions with moderate effect size in psychological distress (Cohen *d*=0.73, 95% CI 0.14-1.32), fear of cancer recurrence (Cohen *d*=0.60, 95% CI 0.02-1.18), rumination (Cohen *d*=0.51, 95% CI 0.09-1.11), and fatigue (Cohen *d*=0.71, 95% CI 0.13-1.29). The analyses also showed improvements with large effect size in mindfulness skills (Cohen *d*=0.81, 95% CI 0.21-1.41), decentering (Cohen *d*=0.81, 95% CI 0.21-1.41), and self-compassion (Cohen *d*=0.73, 95% CI 0.10-1.36). Additionally, well-being was improved, though to a lesser extent (Cohen *d*=0.22, 95% CI 0.32-0.77).

For individual-unguided eMBCT, psychological distress (Cohen *d*=0.83, 95% CI 0.24-1.42) and rumination (Cohen *d*=0.44, 95% CI 0.16-1.04) showed improvements with large ES, while fear of cancer recurrence (Cohen *d*=0.44, 95% CI 0.18-1.07) and fatigue (Cohen *d*=0.46, 95% CI 0.15-1.07) demonstrated smaller effects. Mindfulness skills (Cohen *d*=0.69, 95% CI 0.08-1.30), decentering (Cohen *d*=0.09, 95% CI 0.46-0.65), and self-compassion (Cohen *d*=0.47, 95% CI 0.13-1.07) were also enhanced with medium to large effect sizes (Multimedia Appendix 1).

A third model, including both intervention conditions, showed that the interaction between time and intervention condition was not significant for any of the outcomes, indicating that the degree of change from baseline to follow-up was similar across the 2 delivery formats.

### Prediction

Table 3 and Table S1 in Multimedia Appendix 1 show the results of the prediction and moderation analyses of psychological distress and dropout, respectively. Regarding psychological distress, several baseline variables were identified as significant predictors over time, irrespective of the eMBCT format. Higher rumination, as well as lower mindfulness skills and self-compassion at baseline, predicted a greater reduction in psychological distress from baseline to the 9-month follow-up period (Multimedia Appendix 3). Decentering and cancer diagnosis did not predict treatment outcomes over time, nor did age, sex, cancer treatment, or time since diagnosis. Regarding dropout, none of the predictors were identified as significant.

**Table 3.** Relationship between predictor or moderator, intervention condition^a^, and psychological distress over time. The moderators were coded as follows: sex (0=“male” and 1=“female”), cancer treatment (0=“palliative” and 1=“curative”), and cancer diagnosis (0=“breast cancer” and 1=“no breast cancer”).

Predictor or moderator and variables and interaction terms		Estimate	SE	95% CI	*F* test (*df*)	*P* value
Age						
Age		−0.11	0.08	−0.27 to 0.05	2.52 (1, 278)	.11
Age×time		0.04	0.02	−0.00 to 0.08	1.84 (1, 161)	.18
Age×time×intervention		−0.05	0.03	−0.11 to 0.01	3.73 (1, 161)	.07
Sex						
Sex		−2.03	2.18	−6.30 to 2.24	0.56 (1, 271)	.45
Sex×time		−0.51	0.48	−1.45 to 0.43	1.32 (1, 147)	.25
Male×time×intervention		0.24	0.59	−0.92 to 1.40	0.08 (2, 148)	.09
Cancer treatment						
Cancer treatment		−1.41	2.25	−3.00 to 5.81	1.47 (1, 281)	.23
Cancer treatment×time		0.15	0.50	−0.83 to 1.13	1.68 (1, 159)	.19
Cancer treatment×time×intervention		0.58	0.69	−0.79 to 1.95	0.72 (1, 159)	.39
Cancer diagnosis						
No breast cancer		1.56	1.79	−1.95 to 5.07	4.65 (1, 275)	.03
No breast cancer×time		−0.08	0.40	−1.58 to −0.01	2.02 (1, 155)	.16
No breast cancer×time×intervention		−0.61	0.54	−1.67 to 0.45	1.27 (1, 155)	.26
Time since diagnosis						
Time since diagnosis		0.16	0.14	−0.11 to 0.43	0.16 (1, 277)	.69
Time since diagnosis×time		0.02	0.03	−0.03 to 0.07	0.88 (1, 157)	.35
Time since diagnosis×time×intervention		0.00	0.04	−0.08 to 0.08	0.00 (1, 157)	.95
Rumination (RRQ^b^)						
T0 RRQ		3.06	1.35	0.41 to 5.71	15.09 (1, 258)	<.001
T0 RRQ×time		−0.28	0.33	−0.92 to 0.36	4.17 (1, 155)	.04
T0 RRQ×time×intervention		−0.27	0.41	−1.07 to 0.53	0.42 (1, 155)	.52
Mindfulness skills (FFMQ-SF^c^)						
T0 FFMQ-SF		−0.29	0.08	−0.45 to −0.13	33.31 (1, 256)	<.001
T0 FFMQ-SF×time		0.06	0.02	0.02 to 0.09	10.44 (1, 159)	.00
T0 FFMQ-SF×time×intervention		−0.04	0.03	−0.09 to −0.02	3.02 (1, 159)	.08
Decentering (EQ^d^)						
T0 EQ		−0.29	0.14	−0.56 to −0.02	10.03 (1, 264)	.02
T0 EQ×time		0.05	0.03	−0.10 to 0.00	2.88 (1, 151)	.09
T0 EQ×time×intervention		−0.02	0.04	−0.09 to 0.06	0.23 (1, 151)	.63
Self-compassion (SCS-SF^e^)						
T0 SCS-SF		−0.20	0.07	−0.34 to −0.06	21.75 (1, 259)	<.001
T0 SCS-SF×time		0.04	0.02	0.00 to 0.07	9.87 (1, 143)	.02
T0 SCS-SF×time×intervention		−0.02	0.02	−0.07 to 0.01	0.77 (1, 143)	.38

a Intervention condition was coded as follows: group-blended eMBCT=1 and individual-unguided eMBCT=0.

b RRQ: Rumination and Reflection Questionnaire.

c FFMQ-SF: Five Facet Mindfulness Questionnaire–Short Form.

d EQ: Experiences Questionnaire.

e SCS-SF: Self-Compassion Scale–Short Form.

Binary logistic regression revealed a significant model, including psychological distress and intervention condition. Participants in the group-blended eMBCT were significantly less likely to drop out than those in the individual-unguided format. However, this effect was moderated by psychological distress: within the group-blended condition, participants with higher distress were more likely to drop out compared to those with lower distress. In the individual-unguided condition, psychological distress did not influence dropout rates.

Additionally, results from the linear mixed-effects models showed that none of the 3-way interactions between time, intervention condition, and baseline variables were significant. This suggests that none of the tested baseline characteristics moderated the effect of the eMBCT format on psychological distress over the 9-month follow-up period.

## Discussion

### Principal Findings

This study explores the long-term effects of group-blended and individual-unguided eMBCTs for people with cancer, building on our previous research that focused on short-term (3 months) outcomes in a 3-armed RCT. We now present findings on long-term effects (up to 9 months), including predictors and moderators. As the number of cancer survivors grows, there is an increasing demand for interventions that provide sustained mental health support. Our results show that both eMBCT formats led to lasting improvements in psychological outcomes, reducing distress, fear of recurrence, rumination, and fatigue, while enhancing mindfulness, decentering, and self-compassion. These findings support eMBCT as a scalable, cost-effective approach for addressing the long-term mental health needs of cancer survivors. These findings are consistent with previous research showing that MBIs or eMBIs can have benefits for up to a year in similar populations [13,41]. In addition, beyond reducing symptoms, MBIs have also been shown to support well-being in the long term for people with cancer [6]. Combined with our findings, this evidence underscores MBIs or eMBIs as a meaningful and lasting source of support for this group of individuals.

The results from this study also suggest that individuals with higher levels of rumination or lower levels of mindfulness and self-compassion tended to show greater reductions in distress over time. While those with greater internal resources also experienced improvement, their gains were generally smaller, likely because they started from a more favorable baseline. This suggests that those with more severe initial symptoms may benefit more from eMBCT. These findings are consistent with previous research on standard (in-person) MBCT, which showed that people struggling with depression or anxiety, particularly those who ruminate heavily and have low self-compassion, often experience the most significant improvements [42,43]. Notably, the benefits observed in this research were present for both intervention conditions. This challenges the common assumption that unguided interventions are primarily suitable for individuals with milder symptoms [44].

No differential moderators of treatment effect were identified, suggesting that both eMBCT interventions appear to have similar effects across the diverse sample. However, these findings should be interpreted with caution, given the exploratory nature of the analyses and the potential limitations in statistical power.

### Clinical Implications

As noted, baseline psychological vulnerability (characterized by high rumination, low mindfulness, and low self-compassion) was associated with greater reductions in distress, indicating that those most vulnerable may benefit most from eMBCT. Both group-blended and individual-unguided formats showed comparable effects in reducing psychological distress over the 9-month follow-up, regardless of demographic or clinical factors such as age, sex, cancer type, treatment, or time since diagnosis. Given this, both formats may be broadly offered, enhancing accessibility. While the choice between them can be guided by shared decision-making based on personal preferences, the individual-unguided format may be a more feasible and affordable option when resources are limited.

The dropout rate in the individual-unguided condition was 41%. This aligns with findings from a recent meta-analysis of eMBIs, which reported dropout rates of around 38.7% (95% CI 29.0-49.4) in larger trials [45]. The meta-analysis identified several factors that influenced attrition, such as monetary compensation and the enrollment method, which may partially explain the dropout rates observed in our study. Specifically, we did not offer monetary compensation, and our enrollment process did not involve in-person contact, which previous research has shown can improve retention [45]. Additionally, the higher engagement in the group-blended condition, where participants had personal contact with both the mindfulness teacher and peers, likely fostered a greater sense of accountability, contributing to lower dropout rates in this group. Incorporating more interactive or supportive elements, such as live sessions or compensation strategies, could improve retention in eMBI studies.

The dropout rate was lower in the group-blended eMBCT compared to the individual-unguided format, suggesting that group support may enhance retention. However, within the group-blended condition, participants with higher psychological distress were more likely to drop out. This pattern was not observed in the individual-unguided group. While group formats can offer structure and connection, they may feel overwhelming for individuals in acute distress [46], indicating a need for more tailored or flexible support options.

Despite the higher dropout rate in the individual-unguided format, both versions showed similar effectiveness. It is possible that those who discontinued the unguided format may have derived limited benefit even if they had completed the intervention. This raises the question of whether improving adherence alone is sufficient. Instead, enhancing subjective engagement (how useful participants perceive the intervention to be) may be a more meaningful target for improving outcomes.

As cancer survival rates increase and recurrence remains common [1], long-term support is crucial. MBIs offer practical tools that can be merged into daily life, encouraging habits that promote lasting well-being. Both group-blended and individual-unguided eMBCTs could be implemented and offered by regional or national centers of expertise or networks of qualified mindfulness teachers specialized in psycho-oncological care. Although exploratory, these findings suggest the clinical value of both eMBCTs as scalable interventions that may help people with cancer manage symptoms and maintain psychological well-being over the long term.

### Research Implications

Some important areas for future research are highlighted. First, the absence of assessments of personality traits and user preferences limits our understanding of how individual characteristics may influence engagement with and response to the intervention. For example, traits such as openness, conscientiousness, and neuroticism may impact receptivity to mindfulness practices, adherence to self-guided formats, and overall outcomes. Future studies should incorporate these factors to better tailor and optimize eMBCT for a diverse range of patients.

Second, the study design did not allow us to identify which specific components of eMBCT contributed to the observed outcomes. While the intervention included elements such as guided meditations, homework, and group discussions, their individual roles remain unclear. Further research should investigate the active components of eMBCT to refine and enhance its effectiveness for individuals with varying psychological needs. The eMBCT formats integrate a range of therapeutic elements (eg, guided meditations, reflections, homework, and group discussions), yet their individual or synergistic contributions remain unclear. Understanding the active ingredients of eMBCT is essential for refining and optimizing the intervention, particularly for individuals with specific psychological profiles or needs. Furthermore, designs such as the multiphase optimization strategy or sequential multiple assignment randomized trials could also be used to optimize intervention components, delivery sequences, and intensities for greater personalization and scalability [47].

### Strengths and Limitations

This trial has several key methodological strengths. First, it included a long-term follow-up period, allowing for the assessment of sustained effects. Second, it used 2 eMBCT interventions, enabling a comparison of delivery formats. Third, the group-blended eMBCT was delivered by qualified teachers. Finally, the inclusion of a wide range of outcome measures, beyond psychological distress, provides a holistic understanding of treatment effects. This approach offers valuable insights into both symptom reduction and positive psychological growth.

This study also has several limitations that should be taken into account when interpreting the results. First, the study sample was predominantly female, with nearly half of the participants diagnosed with breast cancer, which means that our findings cannot simply be generalized to other types of cancer. Second, self-selection of participants may have introduced bias, reducing generalizability to less motivated or less digitally inclined cancer survivors. Third, although the primary RCT was adequately powered to detect overall intervention effects from pretreatment to posttreatment, it was not powered to detect small to moderate predictor or moderator effects. Consequently, the analyses were exploratory in nature and involved the examination of a relatively large set of baseline variables without correction for multiple testing; this increases the risk of type I error [48]. Therefore, these findings should be interpreted as hypothesis-generating rather than confirmatory and warrant replication in larger, adequately powered studies. Finally, long-term engagement and how participants integrated mindfulness into their lives posttreatment were not explored.

### Conclusions

Both group-blended and individual-unguided eMBCTs seemed to be effective in reducing psychological distress and enhancing positive mental health outcomes over the course of the intervention and the 9-month follow-up period in people with cancer. Noticeably, individuals with higher rumination and lower mindfulness and self-compassion at baseline showed greater long-term benefits. These findings suggest that both eMBCT formats may have similar effects across the sample, though the absence of significant moderators of treatment effect should be interpreted with caution. The exploratory nature of the analysis and the limited power may have constrained our ability to detect potential moderators. Further studies with larger samples and more robust designs are needed to confirm whether both formats are equally effective for people with cancer. With cost-effectiveness studies underway, our goal is to make these accessible, flexible, and impactful eMBCTs available within the health care system, empowering people with cancer to face their trajectory with greater well-being.

## Supplementary material

10.2196/79928 Multimedia Appendix 1 Statistical analysis plan, supplemental long-term effects figures, and supplemental table on the relationship between the predictors/moderators, intervention condition, and dropout.

10.2196/79928 Multimedia Appendix 2 Participant flowchart showing randomization, completion of intervention conditions, and assessments based on the primary outcome.

10.2196/79928 Multimedia Appendix 3 Supplementary graphs.

## Abbreviations

CAU: care as usual
eMBCT: online mindfulness–based cognitive therapy
eMBI: online mindfulness–based intervention
EQ: Experiences Questionnaire
FFMQ: Five Facet Mindfulness Questionnaire–Short Form
MBCT: mindfulness-based cognitive therapy
MBI: mindfulness-based intervention
RCT: randomized controlled trial
RRQ: Rumination and Reflection Questionnaire
SCS-SF: Self-Compassion Scale–Short Form
