# Supplementary material for: Long-Term Outcomes, Moderators, and Predictors in Online Mindfulness–Based Cognitive Therapy for People With Cancer: Secondary Analysis of a Randomized Controlled Trial
Source: J Med Internet Res. 2026 Apr 17;28:e79928. doi: 10.2196/79928 (PMC13089629; doi:10.2196/79928)
Supplement: Multimedia Appendix 1 [file jmir-v28-e79928-s001.docx]

# Supplementary Material

Contents

Statistical analysis plan (SAP) 2

Amendments to the SAP 7

Long-term effects figures 9

Table S1. Relationship between predictor/moderator, intervention condition, and drop-out12

## Statistical Analysis Plan (SAP) for long-term effects, prediction, and moderation of group-blended and individual-unguided online delivery of mindfulness-based cognitive therapy (eMBCT) for people with cancer

### Administrative Information

### Registration

https://clinicaltrials.gov/ct2/show/NCT05336916

### Protocol

https://bmcpsychology.biomedcentral.com/articles/10.1186/s40359-023-01052-2

### Pilot Study

https://formative.jmir.org/2024/1/e52338

### Introduction

This document contains the data-analysis plan for the consolidation, moderation, and prediction of group-blended and individual-unguided online mindfulness-based cognitive therapy (eMBCT) for people with cancer. People with any type of cancer, at any stage of the disease (including cancer survivors), were randomized to group-blended eMBCT, individual-unguided eMBCT, or care as usual (CAU). Participants initially randomized to CAU were also randomized to either group-blended or individual unguided eMBCT after CAU.

#### Objectives

1. Investigate the *within group* treatment effect of group-blended and individual-unguided eMBCT on the primary (psychological distress) and secondary outcomes (fear of cancer recurrence, fatigue severity, rumination, mindfulness skills, decentering, self-compassion, and well-being) over the course of the nine months follow-up.
2. Explore whether baseline characteristics (sex, age, cancer diagnosis (breast cancer vs other type of cancer), cancer treatment intention, time since diagnosis, rumination, mindfulness skills, decentering, and self-compassion) can predict psychological distress, both in general (predictor) and comparing group-blended and individual unguided eMBCT (moderator).

#### Data Analysis


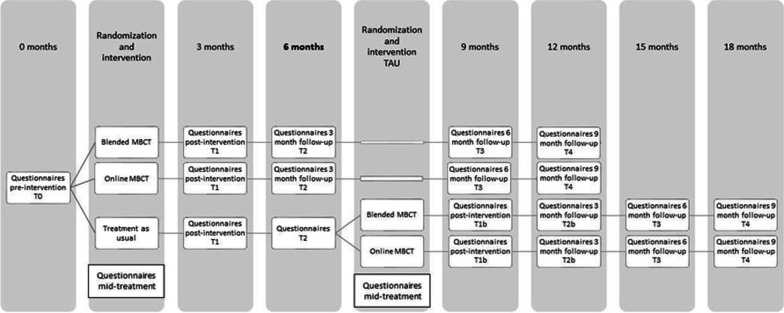
In all analyses, data of participants that followed either group-blended or individual-unguided eMBCT after CAU will be combined with data from participants that were initially randomized to either intervention condition. For the CAU group, T0 scores on the primary and secondary outcome measures will be replaced with T2 scores, as those are closer in time to the start of the intervention. These scores will be compared to the baseline scores of the original group-blended and individual-unguided eMBCT (see image below of time points).

Prior to doing the analyses, assumptions for the linear mixed model will be checked. These assumptions include: collinearity, linearity, homoscedasticity (of residuals), normally distributed residuals, and absence of influential data. In case that one or more assumptions are not met, we will make arrangements to overcome these issues.

#### Objective 1

To answer research question 1, we will build different linear mixed effect models to evaluate long-term effects on our primary and secondary outcomes. We will evaluate whether there is change from post-treatment to the last follow-up within groups. These linear mixed effect models will have the score of each outcome (primary and secondary) as our dependent variable. Independent variables will be *time* (post-treatment, three months follow-up, six months follow-up and nine months follow-up), *group* (group-blended or individual-unguided eMBCT), the *time*group* interaction, and the *baseline measure* (T0) of the corresponding outcome. Time will be treated as a continuous variable. Random intercepts and random slopes for participants will be added. Furthermore, a heterogeneous first-order autoregressive (ARH(1)) covariance structure will be used, which assumes measurements closer in time, are more strongly related. Restricted maximum likelihood (REML) will be used to calculate the estimates.

#### Objective 2

##### Prediction and moderation

Exploratory prediction and moderation analyses of the primary outcome will be conducted using linear mixed models. The following baseline characteristics will be tested as potential predictors/moderators: sex, age, cancer diagnosis, cancer treatment intention, time since diagnosis, rumination, mindfulness skills, decentering, and self-compassion.

The dependent variable will be psychological distress. Independent variables will include:

- Time (post-treatment, three-month follow-up, six-month follow-up, and nine-month follow-up)
- Psychological distress at baseline
- Intervention type (group-blended vs. individual-unguided)
- Baseline characteristics

To comprehensively examine prediction and moderation effects, the following interactions will be included:

1. Baseline characteristic*intervention condition.
2. Time*baseline characteristic.
3. Time*intervention*baseline characteristic.

Random effects will be specified for participants to account for within-subject correlations over time. These models will help determine whether specific baseline characteristics influence intervention effectiveness and how these effects evolve throughout the follow-up period.

## Amendments to the Statistical Analysis Plan

#### The information below outlines the amendments made to the original statistical analysis plan. Key changes are highlighted in red for clarity.

#### Objectives

1. Investigate the effects of group-blended and individual-unguided eMBCT on primary (psychological distress) and secondary outcomes over time from baseline to nine months post-treatment.
2. Explore whether baseline characteristics (sex, age, cancer diagnosis (breast cancer vs other type of cancer), cancer treatment intention, time since diagnosis, psychological distress, rumination, self-compassion, decentering, and mindfulness skills) can predict drop-out, both in general (predictor) and comparing group-blended and individual unguided eMBCT (moderator).

#### Objective 1

We will evaluate whether there is change from baseline to the last follow-up across intervention conditions. Independent variables will be *time* (baseline, post-treatment, three months follow-up, six months follow-up and nine months follow-up).

#### Objective 2

##### Prediction and moderation

The following baseline characteristics will be tested as potential predictors/moderators: sex, age, cancer diagnosis, cancer treatment intention, time since diagnosis, psychological distress, rumination, mindfulness skills, decentering, and self-compassion.

The dependent variable will be psychological distress. Independent variables will include:

- Time (baseline, post-treatment, three-month follow-up, six-month follow-up, and nine-month follow-up)
- Intervention type (group-blended vs. individual-unguided)
- Baseline characteristics (predictors/moderators)

#### Objective 3

Exploratory prediction and moderation analyses of drop-out (coded as 1 = dropped out [< 4 sessions], 0 = completed [≥ 4 sessions]) at post-treatment will be conducted using logistic linear regression models. The following baseline characteristics will be tested as potential predictors and moderators: sex, age, cancer diagnosis, cancer treatment intention, time since diagnosis, psychological distress, rumination, self-compassion, decentering, and mindfulness skills.

The dependent variable will be drop-out status (yes/no) at post-treatment. Independent variables will include:

- Intervention type (group-blended vs. individual-unguided)
- Baseline characteristics (listed above)

To comprehensively examine moderation effects, the following interactions will be tested:

- Baseline characteristic* intervention condition.

## Long-term effects figures

**Fear of Cancer Recurrence**


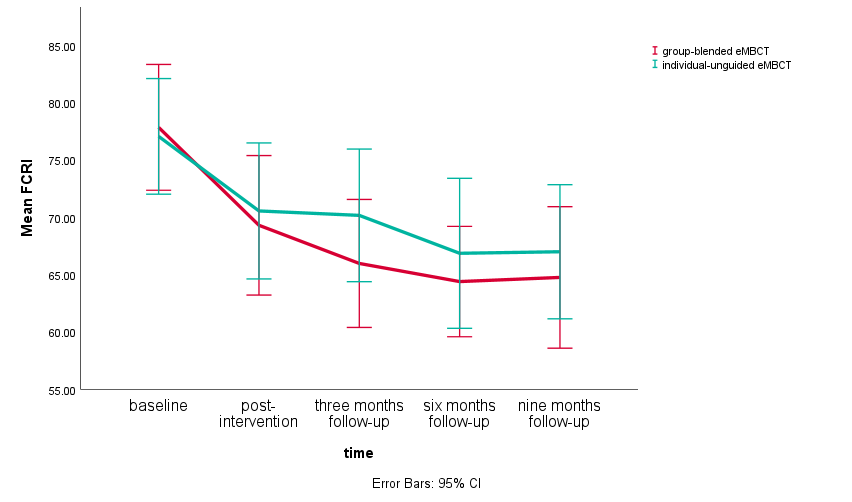


**Mean FCRI**

Figure S1. Change of fear of cancer recurrence (FCRI) over time. Error bars represent 95% confidence intervals.

**Fatigue**


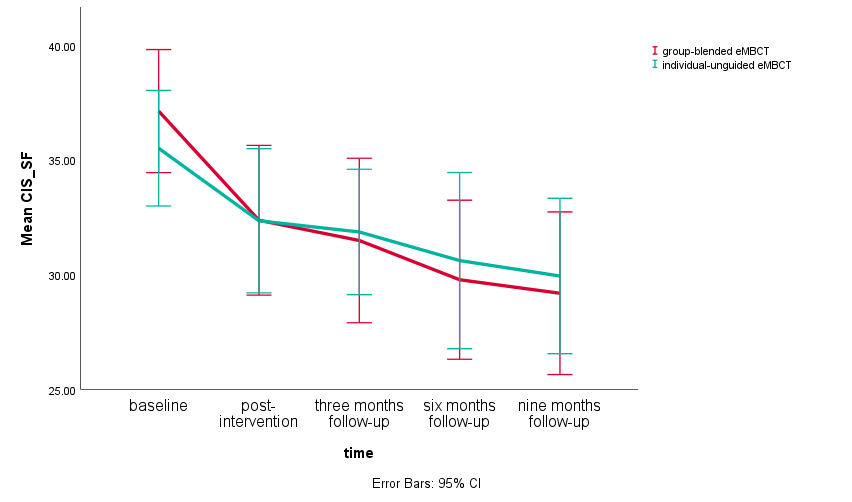


**Mean CIS-F**

Figure S2. Change of fatigue severity (CIS-F) over time. Error bars represent 95% confidence intervals.

**Rumination**


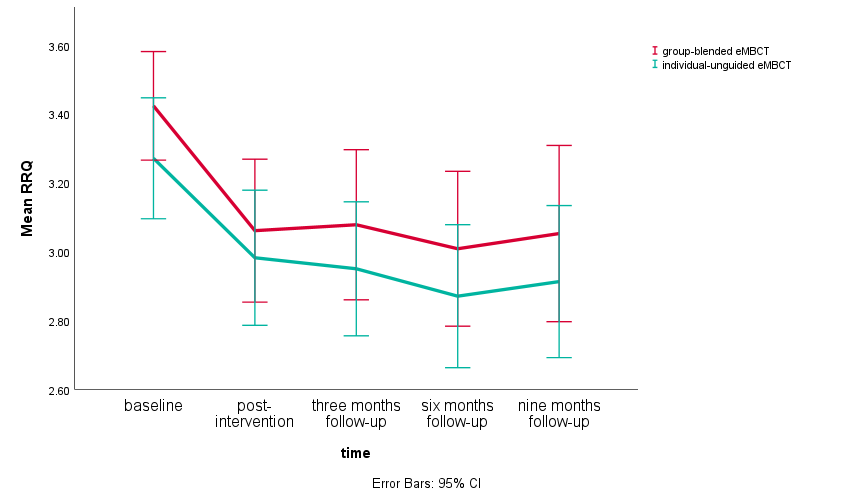


**Mean RRQ**

Figure S3. Change of rumination (RRQ) over time. Error bars represent 95% confidence intervals.

**Mindfulness Skills**


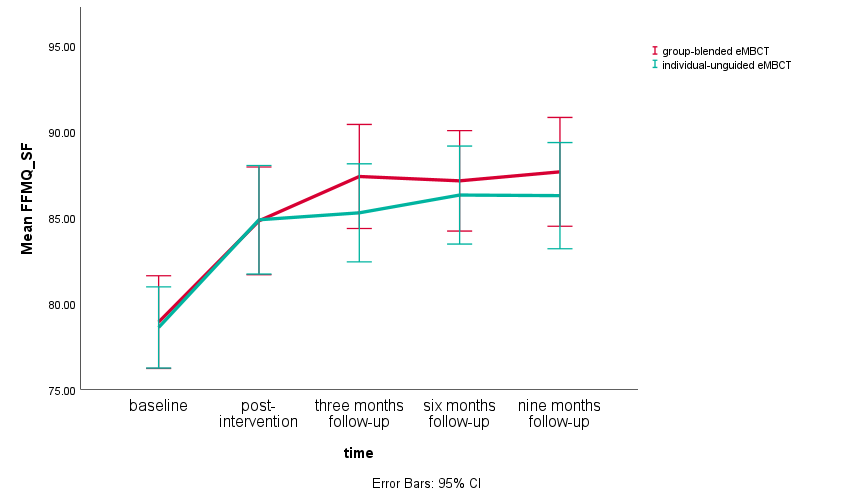


**Mean FFMQ-SF**

Figure S4. Change of mindfulness skills (FFMQ-SF) over time. Error bars represent 95% confidence intervals.

**Decentering**


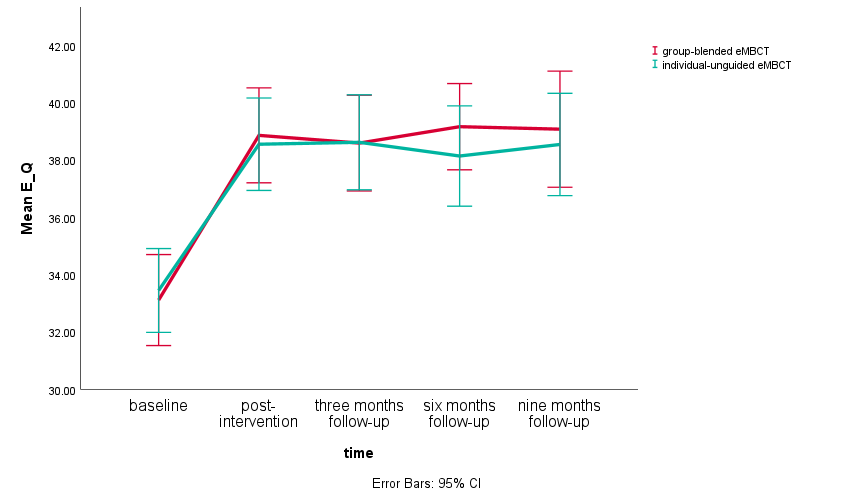


**Mean EQ**

Figure S5. Change of decentering (EQ) over time. Error bars represent 95% confidence intervals.

**Self-Compassion**


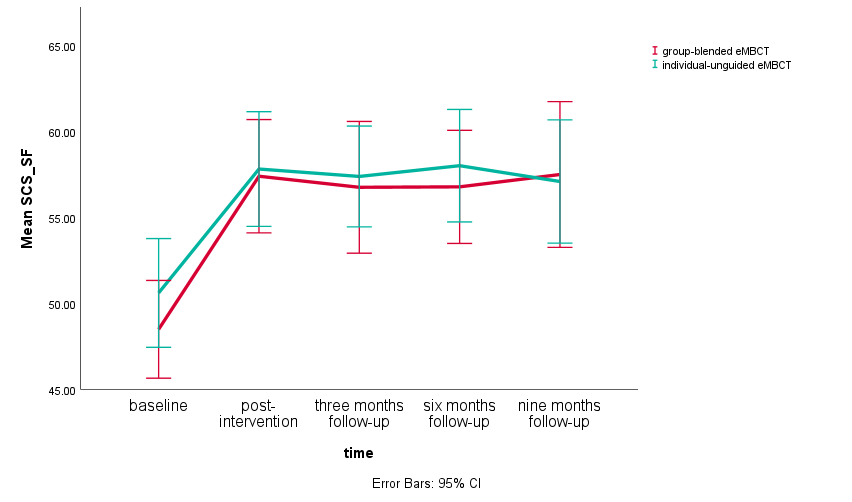


**Mean SCS-SF**

Figure S6. Change of self-compassion (SCS-SF) over time. Error bars represent 95% confidence intervals.

**Well-being**


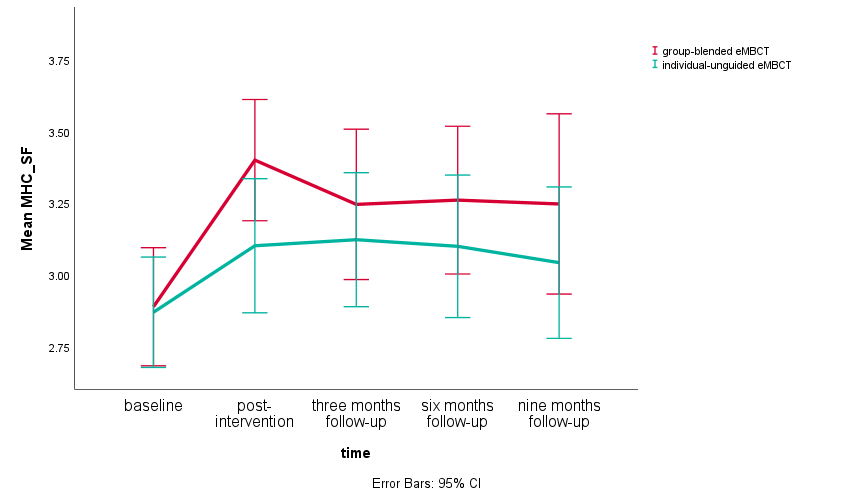


**Mean MHC-SF**

Figure S7. Change of wellbeing (MHC-SF) over time. Error bars represent 95% confidence intervals.

|  |  | Estimate | SE | p |
| --- | --- | --- | --- | --- |
| Intervention condition | Intervention condition | -1.71 | 0.46 | **<0.001** |
| Age | Age | -0.02 | 0.02 | 0.26 |
|  | Age*Intervention | -0.02 | 0.04 | 0.64 |
| Sex | Sex | -0.24 | 0.56 | 0.67 |
|  | Sex*Intervention | 1.95 | 1.11 | 0.99 |
| Cancer treatment | Cancer treatment | -0.28 | 0.54 | 0.60 |
|  | Cancer treatment *Intervention | 0.69 | 1.25 | 0.58 |
| Cancer diagnosis | No breast cancer | -0.79 | 0.45 | 0.08 |
|  | No breast cancer * Intervention | 0.57 | 0.93 | 0.53 |
| Time since diagnosis | Time since diagnosis | -0.13 | 0.07 | 0.07 |
|  | Time since diagnosis *Intervention | 0.07 | 0.12 | 0.57 |
| Psychological distress (HADS) | T0 HADS | -0.00 | 0.03 | 0.94 |
|  | T0 HADS*Intervention | 0.17 | 0.07 | **0.03** |
| Rumination (RRQ) | T0 RRQ | -0.16 | 0.27 | 0.56 |
|  | T0 RRQ*Intervention | -0.29 | 0.64 | 0.65 |
| Mindfulness skills (FFMQ-SF) | T0 FFMQ-SF | 0.01 | 3.28 | 0.79 |
|  | T0 FFMQ-SF *Intervention | -0.03 | 0.42 | 0.44 |
| Decentering (EQ) | T0 EQ | 0.02 | 2.27 | 0.78 |
|  | T0 EQ*Intervention | -0.03 | 0.07 | 0.63 |
| Self-compassion (SCS-SF) | T0 SCS-SF | -0.02 | 0.02 | 0.83 |
|  | T0 SCS-SF*Intervention | -0.03 | 0.04 | 0.44 |

**Table S1. Relationship between predictor/moderator, intervention condition^a^, and drop-out.**

The following moderators were coded as: sex 0=male, 1=female; cancer treatment 0=palliative, 1=curative; cancer diagnosis 0= breast cancer, 1= no breast cancer.

^a^Intervention condition was coded as: group-blended eMBCT=1, individual-unguided eMBCT=0.
