# Supplementary material for: Long-Term Outcomes, Moderators, and Predictors in Online Mindfulness–Based Cognitive Therapy for People With Cancer: Secondary Analysis of a Randomized Controlled Trial
Source: J Med Internet Res. 2026 Apr 17;28:e79928. doi: 10.2196/79928 (PMC13089629; doi:10.2196/79928)
Supplement: Multimedia Appendix 2 [file jmir-v28-e79928-s002.docx]

Participant flow chart showing randomization, completion of intervention conditions, and assessments based on the primary outcome.

Completed T0 (n=35)^a^

Completed T1 (n=54, 72%)

Completed T2 (n=53, 70%)

Completed T3 (n=44, 58%)

Completed T4 (n=44, 58%)

Completed CAU (n=36, 67%)

Assessed for eligibility (n=304)

Randomized (n=186)

Group-blended (n=57)

Individual-unguided (n=75)

CAU (n=54)

Completed T1 (n=43, 75%)

Completed T2 (n=43, 75%)

Completed T3 (n=40, 70%)

Completed T4 (n=34, 60%)

Completed T0 (n=57, 100%)

Completed T0 (n=75, 100%)

Completed T0 (n=54, 100%)

Group-blended (n=14)

Individual-unguided (n=15)

Completed T1 (n=8, 57%)

Completed T2 (n=8, 57%)

Completed T3 (n=10, 71%)

Completed T4 (n=9, 64%)

Completed T1 (n=11, 73%)

Completed T2 (n=13, 87%)

Completed T3 (n=9, 60%)

Completed T4 (n=9, 60%)

Not re-randomized (n=6)

-Did not reply (n=4)

-No interest/other commitments (n=2)

^a^Post-CAU measurement (scores that in the RCT were T2), not all participants were re-randomized.
