# Supplementary material for: Long-Term Outcomes, Moderators, and Predictors in Online Mindfulness–Based Cognitive Therapy for People With Cancer: Secondary Analysis of a Randomized Controlled Trial
Source: J Med Internet Res. 2026 Apr 17;28:e79928. doi: 10.2196/79928 (PMC13089629; doi:10.2196/79928)
Supplement: Multimedia Appendix 3 [file jmir-v28-e79928-s003.docx]

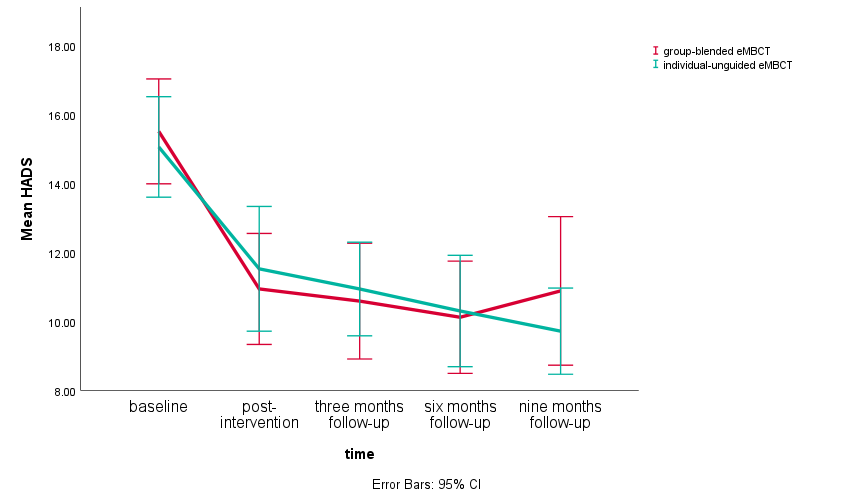


**Psychological Distress**

**Psychological distress (mean HADS)**

Figure S1. Change of psychological distress (HADS) over time for group-blended and individual-unguided eMBCT. Error bars represent 95% confidence intervals.

**Relationship between baseline rumination and change in psychological distress over time**


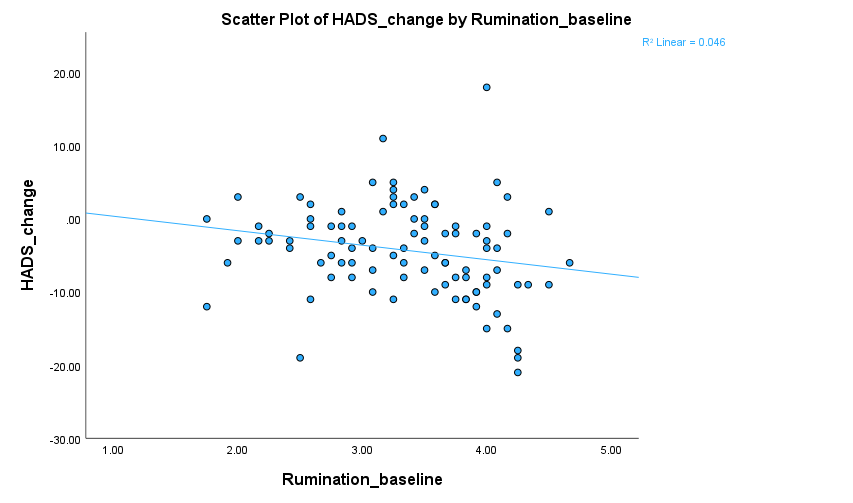


**Change in psychological distress**

**Rumination (baseline)**

Figure S2. Higher rumination at baseline is associated with higher changes in psychological distress over time (from baseline to last (nine months) follow-up). Figure 3 shows that people with higher rumination scores at baseline experience greater reduction in psychological distress over time than people with lower rumination scores at baseline.


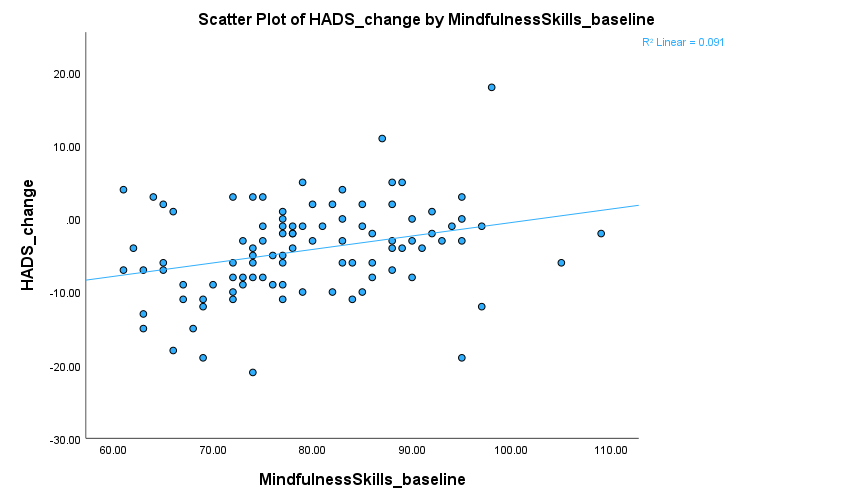


**Change in psychological distress**

**Mindfulness-skills (baseline)**

**Relationship between baseline mindfulness skills and change in psychological distress over time**

Figure S3. Lower mindfulness skills at baseline is associated with higher changes in psychological distress over time (from baseline to last (nine months) follow-up). Figure 4 shows that people with lower mindfulness skills scores at baseline experience greater reduction in psychological distress over time than people with higher mindfulness skills scores at baseline.

**Relationship between baseline self-compassion and change in psychological distress over time**


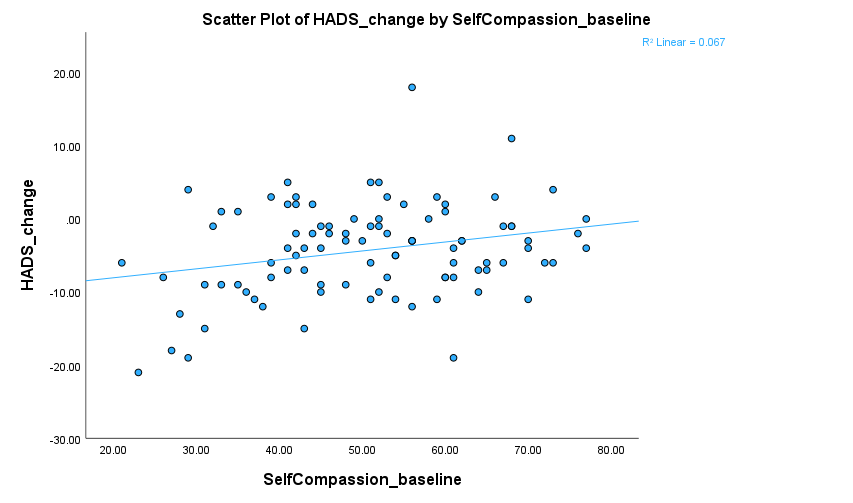


**Change in psychological distress**

**Self-compassion (baseline)**

Figure S4. Lower self-compassion at baseline is associated with higher changes in psychological distress over time (from baseline to last (nine months) follow-up). Figure 5 shows that people with lower self-compassion scores at baseline experience greater reduction in psychological distress over time than people with higher self-compassion scores at baseline
